# The Preventive Health Professions in Italy: The Efficient Use of Resources, Skills and Best Practice during the Pandemic

**DOI:** 10.3390/healthcare10101906

**Published:** 2022-09-28

**Authors:** Vincenzo Marcotrigiano, Fabio Pattavina, Lorenzo Blangiardi, Gerardo Salerno, Annamaria Dalena, Flavio Del Bianco, Marcella Di Fant, Anna Fabbro, Mariarita Forgiarini, Carola Lanzilotti, Malgorzata Wachocka, Paola Marchet, Mirko Mazzurana, Roberto Rizzi, Carmela Russo, Fabiana Salerno, Mattia Vailati, Giacomo Domenico Stingi, Patrizia Laurenti, Antonio Ferro, Sandro Cinquetti, Christian Napoli

**Affiliations:** 1 Prevention Department, Local Health Authority BT, Barletta-Andria-Trani, 76125 Trani, Italy; 2 Hygiene Hospital Unit, Fondazione Policlinico Universitario A. Gemelli IRCCS, 00168 Rome, Italy; 3 Prevention Department, Local Health Authority “AULSS 6 Euganea”, 35131 Padua, Italy; 4 Department of Neurosciences, Mental Health and Sensory Organs “NESMOS”, Sapienza University of Rome, 00185 Rome, Italy; 5 Prevention Department, Local Health Authority Taranto, 74121 Taranto, Italy; 6 Prevention Technical Platform, “AS FO” Western Friuli Health Authority, 33170 Pordenone, Italy; 7 Prevention Department, “ASU FC” Friuli Centrale University Health Authority, 33100 Udine, Italy; 8 Prevention Department, Local Health Authority Brindisi, 72100 Brindisi, Italy; 9 Prevention Department, Local Health Authority “AULSS 1 Dolomiti”, 32100 Belluno, Italy; 10 Prevention Department, Provincial Authority for Health Services, “APSS” Autonomous Province of Trento, 38123 Trento, Italy; 11 Bachelor’s Course in Health Assistance, University of Padua, 35122 Padua, Italy; 12 Authorization for the Accreditation of Healthcare Structures Unit, “ATS” Agency for Health Protection of Metropolitan Area of Milan, 20122 Milan, Italy; 13 Life Sciences and Public Health Department, Università Cattolica del Sacro Cuore, 00168 Rome, Italy; 14 Department of Medical Surgical Sciences and Translational Medicine, Sapienza University of Rome, 00189 Rome, Italy

**Keywords:** public health, pandemic response, health visitors, environmental health officers, multi-professional, inter-professionalism, prevention departments

## Abstract

Health visitors (HVs) and environmental health officers (EHOs) are the healthcare workers (HCWs) who, in the Italian National Health Service, mainly operate in the prevention departments of local health authorities, guaranteeing the territorial activities specifically declared with the respective professional profiles. During the SARS-CoV-2 pandemic, it was necessary to reallocate all HCWs supporting Hygiene and Public Health Services involved on the front lines of the emergency, in order to perform preventive activities and to take immediate action to fight the spread of the virus. By means of an IT survey consisting of three sections, this study investigated how 960 HVs and EHOs dealt with this reallocation, with the shifting in service assignment, and with the perceived level of fatigue and pressure, through the application of skills acquired from university training. The synergy among the preventive health professions, the ability to work in a multi-professional team, and the complementary training of HCWs represent the main strengths for overcoming future public health challenges, aimed at protecting human health.

## 1. Introduction

Over the last few decades, the Italian National Health Service (INHS) has invested heavily in specialist diagnostics, therapies, and interventions to treat acute and chronic clinical events. Although INHS has achieved considerable clinical success [1], the SARS-CoV-2 and the related difficulty in managing the pandemic emergency also caused significant repercussions to the ability of the system to prevent and treat non-COVID-19 diseases [1,2,3].

Territorial healthcare services, such as prevention departments (PDs), initially highlighted severe difficulties in fighting the pandemic and questioned the ineffectiveness of the territorial organizational system. Because of the need to provide a timely and efficient response to COVID-19 cases, the PDs suddenly had to reassign their own activities and reallocate their professional skills. This new reorganization was partially in line with the principles of the task-shifting model, which can be considered among the most efficient features of the Primary Health Care strategy. The World Health Organization recognizes it as a model for pursuing global health in specific areas or zones [4].

Health visitors (HVs)––in some countries, health assistants or healthcare assistants––and environmental health officers (EHOs) work under territorial assistance and represent the suitable professionals for performing preventive activities such as vaccinations, official controls, health promotion, and training. The respective professional profiles of the HCWs help define the operational lines along which they operate in a complementary and synergistic way.

An HV, according to Italian health legislation, is an HCW who is responsible for health prevention and promotion, and for providing education to people, families, and the community. The HV identifies the priorities for preventive, educational, and recovery intervention and plans and implements health education interventions for people of all ages and campaigns for health promotion and education. The HV also monitors hygienic–sanitary conditions in families, schools, and assisted-living communities as well as the environment for conditions that favor infectious risks [5].

In the same context, the EHO is in charge of prevention, verification, and control activities regarding hygiene in the areas of living and work places, food and beverage, public health, and veterinary hygiene. The EHO monitors and controls living and working environments, the safety of structures and systems, and their regulatory compliance, including the field of environmental prevention. Conducting inspection and surveillance tasks under the local health authority, this professional figure is given the role of a sanitary police officer [6]. These health professionals provided the main contribution to the reorganization of territorial assistance and to the fight against the pandemic during the emergency.

In this context, the aim of this study is to investigate the main features of the working activities of HVs and EHOs compared to the pre-pandemic era. The highlighted strengths and areas for improvement also suggest possible opportunities for professional education and reallocation in emergencies.

## 2. Materials and Methods

### 2.1. Setting and Participants

During the period May–June 2022, two surveys with brief introductions about their objectives were administered to HVs and EHOs using dedicated links accessible via the Google Forms platform. The link was sent to the 235 HVs/EHOs who joined the Italian Society of Hygiene, Preventive Medicine and Public Health (S.It.I.) in 2021. They were simultaneously asked to extend the invitation to colleagues with the same professional role and working at the same place or at other authorities, even if they were not S.It.I. members.

A minimum sample size of at least 338 HV- and 365 EHO-enrolled individuals was required to investigate the selected variables in the surveyed HCW population. The sample was calculated by a sample size calculator, based on the reference population of the two HCW groups and assuming a response rate of 50%, a 95% confidence level and a 5% margin of error.

### 2.2. Questionnaire

The survey comprised an original questionnaire written in Italian consisting of three sections. The first section collected socio-demographic information (i.e., gender, age, and working region of origin). The second section investigated the respondent’s assignment to a PD service, type of employment contract, and perceived level of fatigue and pressure during the pre-pandemic era. The third section investigated the same aspects as the second one, but during the pandemic; to this purpose, additional questions were added with regard to the skills acquired during university training, the need to acquire new or more in-depth knowledge or an additional skill, the anti-SARS-CoV-2 vaccination and any positivity to COVID-19. The questionnaire was tested in a pilot study (data not published), using the same methodology reported in previous studies [7,8].

The measure of the questions’ comprehensibility was evaluated on the pilot study group of 27 people: 12 HVs and 15 EHOs. This pilot group was asked to assign a rating to each question on a scale from 1 (not meaningful) to 7 (very meaningful). A mean score >5 per question was considered to be the cutoff for acceptability. For this purpose, the original questionnaire was modified: aside from questions belonging to the standard questionnaire (SQQ), there were 10 additional questions (AQs) reporting grammatical and semantic errors (e.g., the use of the verb “to be” in place of “to have” and the use of generic words such as “things” in place of specific words) to guarantee answer variability. The standard questionnaire reported a mean score for each question >5; AQs reported a mean score for each question <2. These data confirmed that the content of the questionnaire was clear to the readers. In the same pilot sample, the reliability index for SQQ was assessed using Cronbach’s alpha (internal consistency coefficient), for both the pilot and original study. The alpha values were 0.74 and 0.76 for HVs and EHOs, respectively, which showed satisfactory reliability.

The investigation was performed in accordance with the World Medical Association Declaration of Helsinki and did not include any experiments involving human or biological human samples, nor research on identifiable human data. Nevertheless, the protocol was approved by the S.It.I. Scientific Board with the number 1R0316_2022.

### 2.3. Statistical Analysis

A descriptive analysis was performed on the participants’ demographic characteristics and working experience. Age was expressed as a mean value ± standard deviation (SD). Other characteristics and answers were reported as the numbers and percentages of respondents, while continuous values were expressed with the mean value and corresponding SD. The normality of all the variables in the study was evaluated with the D’Agostino–Pearson normality test. The Chi square test was applied to evaluate the difference in proportions. Differences between the two groups were evaluated by applying the paired Student’s *t*-test. The level of significance for all statistical tests performed was set at *p* < 0.05. All statistical data analyses were carried out with the software R version 4.0.3 (2020-10-10)—(“Bunny-Wunnies Freak Out” Copyright© 2020 The R Foundation for Statistical Computing).

## 3. Results

Overall, 960 HCWs were involved in the survey: 430 HVs (345 women, 80.23%, and 85 men, 19.77%) and 530 EHOs (140 women, 26.41%, and 390 men, 73.59%). Table 1 shows the distribution of responses by geographical area, and Table 2 shows the number of respondents by age group.

It should be pointed out that 90 of 430 HVs (20.93%) and 30 of 530 EHOs (5.66%) declared themselves to have been unemployed in the pre-pandemic period, and that 62 HVs (14.42%) and 53 EHOs (10%) were hired on a temporary contract stipulated by a health emergency (e.g., fixed term or freelance professional). Table 3 shows the respondents’ assigned employment service.

Furthermore, the level of perceived fatigue and pressure was investigated, with respondents asked to assign a value to two specific questions, using a numerical scale between 1 and 10. Results are shown in Table 4.

The results of knowledge and skills are shown in Table 5.

Furthermore, 31 responding HVs (7.21%) and 70 responding EHOs (13.21%) declared that they also practiced smart working. All respondents benefited from the anti-SARS-CoV-2 vaccination and 195 HVs (45.35%) and 212 EHOs (40%) declared that they had contracted SARS-CoV-2 infection.

## 4. Discussion

Before the 2009 influenza A(H1N1) pandemic, most European Union member states had developed preparedness plans to respond to a pandemic in a timely manner. Many of these plans involved explicit or implicit assumptions on what could be expected, how a pandemic virus might behave, and the role of early warning strategies [9,10,11]. Unfortunately, the non-updated Italian plan was not enough to stop the start of the pandemic in Italy.

In the absence of complete specific and updated guidelines for pandemic events and organizational strategies for maximum emergency management, PD services suddenly had to program important organizational changes according to iso-resources. In the first period, large-scale epidemiological investigations were needed; subsequently, diagnostic tests for case control and confirmation had to be guaranteed; later, PDs organized and ensured a mass vaccination campaign, which initially involved HCWs as a priority [11]. This affected numerous organizational and PD managerial aspects. In fact, our study showed a statistically significant increase in the number of HCWs assigned to Hygiene and Public Health Services during the pandemic because of the need to reallocate human resources to the front lines of the emergency. The HVs enrolled in our study were significantly younger than the EHOs, probably because the pandemic gave an impetus to hiring dedicated HCWs to manage epidemiological data and vaccination campaigns. These professionals, often recent graduates, found immediate placement in the Hygiene and Public Health Services of the PDs.

A significant difference emerged from the responses provided by both groups of HCWs concerning the perception of excessive fatigue or the lack of sufficient rest time. Both HVs and EHOs stated that they perceived a more excessive level of pressure during the pandemic, which reflected how the heavier workloads increased the perceived risk of causing errors, accidents and disorders. Our results are in line with those in the literature, which found that during the pandemic, psychological distress, sleep, and mental disorders were prevalent among HCWs [12], although sleep disorders were also found in the general population [13]. Anxiety and burnout were also described in HCW studies [14,15], to the point where the quality of professional life and personal identity were compromised [16].

It is useful to highlight that more than 50% of the HVs interviewed said the skills acquired at university were sufficient for the full scope of routine and non-emergency work activities, whereas almost 80% of the EHOs did not consider their university skills sufficient. This should be considered as a starting point for improvement to continue vividly encouraging, at University level, interventions aimed at enhancing basic, transversal and technical–professional skills through dedicated extra courses, if necessary. By means of the laboratories of community of practice, the sharing of experiences, and the promotion of the use of the most innovative IT, it is possible to satisfactorily continue to train the professionals of tomorrow. A re-evaluation of some aspects of university training may emerge from this survey, although the age of the respondents may be a confounding factor: in our study, HVs were statistically younger than EHOs, and in recent years the core curriculum of HCW degree courses has radically changed. Nevertheless, it would be appropriate to investigate the study paths of preventive health professionals given that the skills acquired from university courses are not considered sufficient for HVs or EHOs to deal with a specific emergency. Public health best practices teach that leadership and coordination skills, on top of essential theoretical knowledge, are necessary for adequate emergency management [17].

The HVs and EHOs interviewed declared that in the pandemic they needed new or more in-depth knowledge and skills, even if these were not strictly related to their professional profile. This response is related to the benefits and liabilities of increasing computerization and information, which created the “infodemic” phenomenon, one of the new challenges of public health [18].

Within the Italian National Recovery and Resilience Plan, Mission 6, “Health” has two components: “innovation, research and digitization of the national health service” and “digital updating and training, scientific research and technology transfer” [19]. The pre-existing National Prevention Plan (PNP), 2014–2018, identified the central support line A.1.3—“More efficient use of professional resources: hypothesis of Task Shifting”—designed to study possible integration among professionals with particular attention to prevention, early diagnosis, and health promotion interventions [20]. The PNP 2020–2025, continuing the previous one, underlines the indispensability of health planning based on a coordinated and integrated network among territorial structures and activities. In this context, the flexibility of health professionals assigned to the service is central because it supports the organizational, functional, and operational interaction of all internal and external resources in the health system and the PD [21]. As a rule, HCWs such as hygienists and occupational physicians, veterinarians, HVs, EHOs, and in some contexts also nurses and dieticians, work in the Italian PDs, guaranteeing collective prevention, environmental risk evaluation, and public health functions, contributing to health promotion and non-communicable disease prevention [22,23,24].

The Italian PDs have already started this process by having all available HCWs work synergistically. This was demonstrated during the pandemic concerning epidemic intelligence promotion, internal processes reorientation, COVID-19 case management, and specific training and continuous education (e.g., contact tracing and the correct use of personal protective equipment) [25]. On the subject of risk assessment and nosocomial risk management, it was necessary to implement immediate anti-contagion protocols to safely contain the pandemic to ensure the protection of patients and hospital staff [26,27,28], and to continue promoting safe behavior; also, in order to fight breakthrough infections [29]. Furthermore, with reference to territorial control activities, numerous HCWs provided support to schools [30], businesses, private companies, and associations following prevention protocols, as well as interservice inspections to jointly assess the risks at the local level [31]. In addition, specific field studies were conducted to investigate the spread of SARS-CoV-2 in life and work settings to allow for a more appropriate risk assessment [32,33,34]. This was also possible because of the synergy among HCWs, and during training courses [35,36,37].

Unfortunately, the efforts of HVs and EHOs have not yet received full appreciation either from the media or institutions. Nevertheless, HCWs are constantly exceeding expectations for community service. The lesson here is that it is necessary to continue to support and raise awareness of the need to make territorial services more efficient among health management. To date, scientific evidence has identified the principles of task shifting as a concrete support for improving the prevention system, especially in developing countries [38,39].

The authors are aware of some limitations to the study. First, the variables were not investigated in depth to avoid an excessively long questionnaire, but this could have prevented the collection of important information. Furthermore, this study explored a specific group of HCWs (HVs/EHOs) that is not representative of the entirety of Italy’s HCW population. Moreover, the sample showed a higher component of HCWs from northern and southern regions, which probably reflected the sampling procedure and compliance with the investigation of some regions. Therefore, this study can be considered a preliminary research. Nevertheless, during a period of emergency, it may help address future public information campaigns earlier and highlight the need to emphasize the importance of improving territorial health service organization and to promote the professionals in PDs [40].

## 5. Conclusions

To the best of our knowledge, this is the first study to assess changes in the working activities and preparedness levels of HCWs in the prevention field in Italy. Numerous strengths emerged from this experience: the unitary organization of healthcare professions with methods aimed at enhancing their skills, sharing objectives, and balancing available resources, together with a marked propensity for constructive integration among professionals [41]. The prospects for multidisciplinary growth can be initiated by developing priority alliances among preventive health professions with other professionals involved in institutional activities that provide preventive services to protect the community and to promote public health.

## Figures and Tables

**Table 1:** Origin of answers provided by geographic area.

Geographic Area of Response	HV Respondents (%)	EHO Respondents (%)	*p*-Value
Northern Italy	229 (53)	226 (43)	**0.0130**
Central Italy	18 (4)	65 (12)	**<0.0001**
South Italy	183 (43)	239 (45)	0.4703
	430 (100)	530 (100)	

**Table 2:** Number of respondents by age group.

	HVs	EHOs	
Age Range	*n* (%)	*n* (%)	*p*-Value
≤30	171 (39.77)	110 (20.75)	<0.0001
31–40	154 (35.81)	303 (57.17)	<0.0001
41–50	39 (9.07)	26 (4.91)	0.0153
51–60	66 (15.35)	57 (10.76)	0.0433
≥61	0	34 (6.41)	<0.0001
	430 (100)	530 (100)	

**Table 3:** Respondent assignment services in pandemic and pre-pandemic eras.

	HVs			EHOs		
Assignment Service	Pre-Pandemic *n* (%)	Pandemic *n* (%)	*p*-Value	Pre-Pandemic *n* (%)	Pandemic *n* (%)	*p*-Value
Hygiene and Public Health Service	176 (40.93)	330 (76.74)	<0.0001	219 (41.32)	358 (67.55)	<0.0001
Food Hygiene and Nutrition Service	10 (2.32)	4 (0.93)	0.1779	126 (23.78)	55 (10.38)	<0.0001
Workplace Safety Prevention Service	34 (7.91)	30 (6.98)	0.6667	65 (12.26)	62 (11.7)	0.8500
Veterinary Services	0	0	1.0000	10 (1.89)	8 (1.51)	0.8121
Other Prevention Department Services: Health and Environment, Promotion of Physical Activities, Epidemiology Services	47 (10.93)	31 (7.21)	0.0749	12 (2.26)	0	0.0014
Services not included in the Department of Prevention: Prevention and Protection Services, Technical Services, Environmental Protection Agencies, Border Control Posts, Universities	73 (16.98)	35 (8.14)	<0.0001	68 (12.83)	47 (8.86)	0.0482
No employment in pre-pandemic era	90 (20.93)	0	<0.0001	30 (5.66)	0	<0.0001
	430 (100)	430 (100)		530 (100)	530 (100)	

**Table 4:** Fatigue and pressure perceived by HVs and EHOs in pre-pandemic and pandemic era.

	HVs			EHOs		
	Pre-Pandemic Mean (SD)	Pandemic Mean (SD)	*p*-Value	Pre-Pandemic Mean (SD)	Pandemic Mean (SD)	*p*-Value
Working activities in which you are involved requiring fatigue or not guaranteeing sufficient rest times	6.14 (2.31)	7.69 (2.50)	<0.0001	6.4 (2.11)	7.34 (2.35)	<0.0001
Working activities, combined with any shifts, exposing you to an excessive level of pressure	5.94 (2.40)	8.02 (2.89)	<0.0001	5.89 (2.49)	7.39 (2.59)	<0.0001

**Table 5:** Knowledge and skills declared in the survey.

	HVs			EHOs		
Knowledge and Skills Area	Yes (%)	No (%)	*p*-Value	Yes (%)	No (%)	*p*-Value
Were the skills acquired in your university career sufficient for carrying out your work activities with full autonomy?	234 (54.42)	196 (45.58)	0.0116	110 (20.75)	420 (79.25)	<0.0001
Were the skills acquired in your university career sufficient for carrying out the work specifically executed in the emergency context?	185 (43.02)	245 (56.98)	0.0001	108 (20.38)	422 (79.62)	<0.0001
In the pandemic period, did you need to acquire new or more in-depth knowledge or additional skills, even if not strictly related to your professional profile?	353 (82.09)	77 (17.91)	<0.0001	505 (95.28)	25 (4.72)	<0.0001

## Data Availability

Not applicable.

## References

[1] (2021). ISTAT Rapporto Annuale del Paese. https://www.istat.it/storage/rapporto-annuale/2021/Rapporto_Annuale_2021.pdf.

[2] Piane M., Bianco L., Mancini R., Fornelli P., Gabriele A., Medici F., Battista C., Greco S., Croce G., Franceschetti L. (2022). Impact of the COVID-19 Pandemic on Clinical Pathways for Non-SARS-CoV-2 Related Diseases in the Lazio Region, Italy. Int. J. Environ. Res. Public Health.

[3] Di Stefano V., Rispoli M.G., Pellegrino N., Graziosi A., Rotondo E., Napoli C., Pietrobon D., Brighina F., Parisi P. (2020). Diagnostic and therapeutic aspects of hemiplegic migraine. J. Neurol. Neurosurg. Psychiatry.

[4] World Health Organization (2008). The World Health Report 2008—Primary Health Care (Now More Than Ever).

[5] Decreto Ministeriale 17 gennaio 1997, n. 69. Regolamento concernente la individuazione della figura e relativo profilo professionale dell’assistente sanitario.

[6] Decreto Ministeriale 17 gennaio 1997, n. 58. Regolamento concernente la individuazione della figura e relativo profilo professionale del tecnico della prevenzione nell’ambiente e nei luoghi di lavoro.

[7] Gallé F., Quaranta A., Napoli C., Diella G., De Giglio O., Caggiano G., Di Muzio M., Stefanizzi P., Orsi G.B., Liguori G. (2022). How do Vaccinators Experience the Pandemic? Lifestyle Behaviors in a Sample of Italian Public Health Workers during the COVID-19 Era. Vaccines.

[8] Marcotrigiano V., Stingi G.D., Fregnan S., Magarelli P., Pasquale P., Russo S., Orsi G.B., Montagna M.T., Napoli C., Napoli C. (2021). An Integrated Control Plan in Primary Schools: Results of a Field Investigation on Nutritional and Hygienic Features in the Apulia Region (Southern Italy). Nutrients.

[9] Napoli C., Fabiani M., Rizzo C., Barral M., Oxford J., Cohen J., Niddam L., Goryński P., Pistol A., Lionis C. (2015). Assessment of human influenza pandemic scenarios in Europe. Eurosurveillance.

[10] Napoli C., Riccardo F., Declich S., Dente M.G., Pompa M.G., Rizzo C., Rota M.C., Bella A., National Working Group (2014). An early warning system based on syndromic surveillance to detect potential health emergencies among migrants: Results of a two-year experience in Italy. Int. J. Environ. Res. Public Health.

[11] Dettori M., Arghittu A., Deiana G., Azara A., Masia M.D., Palmieri A., Spano A.L., Serra A., Castiglia P. (2021). Influenza Vaccination Strategies in Healthcare Workers: A Cohort Study (2018–2021) in an Italian University Hospital. Vaccines.

[12] Pisanu E., Di Benedetto A., Infurna M.R., Rumiati R.I. (2022). Psychological Impact in Healthcare Workers during Emergencies: The Italian Experience with COVID-19 First Wave. Front. Psychiatry.

[13] Gallè F., Sabella E.A., Roma P., Ferracuti S., Da Molin G., Diella G., Montagna M.T., Orsi G.B., Liguori G., Napoli C. (2021). Knowledge and Lifestyle Behaviors Related to COVID-19 Pandemic in People over 65 Years Old from Southern Italy. Int. J. Environ. Res. Public Health.

[14] Ghahramani S., Kasraei H., Hayati R., Tabrizi R., Marzaleh M.A. (2022). Health care workers’ mental health in the face of COVID-19: A systematic review and meta-analysis. Int. J. Psychiatry Clin. Pract..

[15] Orrù G., Marzetti F., Conversano C., Vagheggini G., Miccoli M., Ciacchini R., Panait E., Gemignani A. (2021). Secondary Traumatic Stress and Burnout in Healthcare Workers during COVID-19 Outbreak. Int. J. Environ. Res. Public Health.

[16] Caricati L., D’Agostino G., Sollami A., Bonetti C. (2022). A study on COVID-19-related stigmatization, quality of professional life and professional identity in a sample of HCWs in Italy. Acta Biomed..

[17] Laus F. (2022). Can the emergency response be coordinated?. Int. J. Risk Saf. Med..

[18] Briand S.C., Cinelli M., Nguyen T., Lewis R., Prybylski D., Valensise C.M., Colizza V., Tozzi A.E., Perra N., Baronchelli A. (2021). Infodemics: A new challenge for public health. Cell.

[19] (2022). Italian National Recovery and Resilience Plan. https://italiadomani.gov.it/it/home.html.

[20] Faggiano F., Pirastu R., Allara E., Falcone M., Ferrante G., Pacelli B., Schifano P., Senore C., Serinelli M. (2015). Epidemiology and prevention at the times of the Italian National Prevention Plan 2014–2018. Epidemiol. Prev..

[21] Italian Ministry of Health, National Prevention Plan 2020–2025. https://www.salute.gov.it/imgs/C_17_notizie_5029_0_file.pdf.

[22] Decreto Legislativo 19 giugno 1999, n. 229. Norme per la razionalizzazione del Servizio sanitario nazionale, a norma dell’articolo 1 della legge 30 novembre 1998, n. 419.

[23] Montagna M.T., De Giglio O., Cristina M.L., Napoli C., Pacifico C., Agodi A., Baldovin T., Casini B., Coniglio M.A., D’Errico M.M. (2017). Evaluation of Legionella Air Contamination in Healthcare Facilities by Different Sampling Methods: An Italian Multicenter Study. Int. J. Environ. Res. Public Health.

[24] Napoli C., Iatta R., Fasano F., Marsico T., Montagna M.T. (2009). Variable bacterial load of *Legionella* spp. in a hospital water system. Sci. Total Environ..

[25] Mantovani W., Franchini S., Mazzurana M., Zuccali M.G., Pizzo F., Zanin A., Ferro A., Gruppo Segnalazioni del Dipartimento di Prevenzione APSS (2020). Reorganization and public health management by the Department of Prevention during the COVID-19 emergency. An experience of integration between prevention and primary care in the proactive management of possible cases. Epidemiol. Prev..

[26] Wachocka M., Pattavina F., Palluzzi V., Cerabona V., Laurenti P. (2020). Health Professionals of Prevention in Italy: The Value of Expertise During COVID-19 Pandemic. Front. Public Health.

[27] Lampedecchia M., Marcotrigiano V., Sorrenti G.T., Magarelli P., Stoppato U., Albergo F. (2021). Riorganizzazione dell’attività lavorativa dell’operatore sanitario nel contesto emergenziale da SARS-CoV-2: Analisi dei pericoli e prospettive future. J. Prev. Med. Hyg..

[28] Jachetti A., Colombo G., Brignolo-Ottolini B., Franchi J., Solbiati M., Pecorino Meli M., Bosco P., Costantino G. (2021). Emergency department reorganisation to cope with COVID-19 outbreak in Milan university hospital: A time-sensitive challenge. BMC Emerg. Med..

[29] Porru S., Monaco M.G.L., Spiteri G., Carta A., Pezzani M.D., Lippi G., Gibellini D., Tacconelli E., Dalla Vecchia I., Sala E. (2022). SARS-CoV-2 Breakthrough Infections: Incidence and Risk Factors in a Large European Multicentric Cohort of Health Workers. Vaccines.

[30] Candela G., Del Bianco F., Lo Giudice A., Bolzonello C., Bomben L., Sumelli C., Biasotto E., Pilan S. (2021). The management of confirmed cases of Covid-19 infection in schools: The experience of the prevention Department of the Azienda Sanitaria Friuli occidentale in the Pordenone area. Ig. Sanita Pubblica.

[31] Marcotrigiano V., Sorrenti G.T., Sorrenti D.P., Lampedecchia M., Matera R., Stingi G.D., Magarelli P. (2021). Azioni di prevenzione e contrasto alla diffusione di SARS-CoV-2: L’esperienza territoriale interservizi dell’Azienda Sanitaria Locale BT. J. Prev. Med. Hyg..

[32] Montagna M.T., De Giglio O., Calia C., Pousis C., Apollonio F., Campanale C., Diella G., Lopuzzo M., Marzella A., Triggiano F. (2021). First Detection of Severe Acute Respiratory Syndrome Coronavirus 2 on the Surfaces of Tourist-Recreational Facilities in Italy. Int. J. Environ. Res. Public Health.

[33] Caggiano G., Apollonio F., Triggiano F., Diella G., Stefanizzi P., Lopuzzo M., D’Ambrosio M., Bartolomeo N., Barbuti G., Sorrenti G.T. (2021). SARS-CoV-2 and Public Transport in Italy. Int. J. Environ. Res. Public Health.

[34] Caggiano G., Triggiano F., Apollonio F., Diella G., Lopuzzo M., D’Ambrosio M., Fasano F., Stefanizzi P., Sorrenti G.T., Magarelli P. (2021). SARS-CoV-2 RNA and Supermarket Surfaces: A Real or Presumed Threat?. Int. J. Environ. Res. Public Health.

[35] Russo C., Marcotrigiano V. (2015). Assistenti sanitari e tecnici della prevenzione per la Sanità Pubblica e la Prevenzione. Calamo Specchia F. Manuale Critico di Igiene e Sanità Pubblica.

[36] Marcotrigiano V., Russo C., Dall’Armi R., Grillo A., Iuliano G., Cinquetti S., Baldo V. (2014). Formare alla comunità di pratica tra Assistenti Sanitari e Tecnici della Prevenzione (Classe L/SNT4): Un’esperienza con gli studenti del Corso di Laurea in Assistenza Sanitaria. Ann. Ig..

[37] Marcotrigiano V., Fabbro A. (2019). La prevenzione in ambito territoriale: Necessità di valorizzazione in un contesto multiprofessionale. J. Adv. Health Care JAHC.

[38] Gruppo di Lavoro Task Shifting Società Italiana d’Igiene, Medicina Preventiva e Sanità Pubblica. http://www.sitinazionale.org/bds/index.php/gruppi-di-lavoro/task-shifting/linee-guida-e-normative.

[39] Marcotrigiano V. (2019). La valorizzazione dei Professionisti della Prevenzione: Minaccia organizzativa o opportunità di miglioramento?. J. Prev. Med. Hyg..

[40] Torri E., Sbrogiò L.G., Rosa E.D., Cinquetti S., Francia F., Ferro A. (2020). Italian Public Health Response to the COVID-19 Pandemic: Case Report from the Field, Insights and Challenges for the Department of Prevention. Int. J. Environ. Res. Public Health.

[41] Pacetti P. (2022). L’Assistente Sanitario: Un Professionista Per tutti i Servizi Sanitari e Sociali. Manuale Operativo.

